# Saunders’ Hand Atlas

**Published:** 1903-10

**Authors:** 


SAUNDERS' HAND ATLAS—Atlas and Epitome of Otology. By Gustav Bruhl, M. D., of Berlin, with the collaboration of Prof. Dr. A. Politzer, of Vienna. Authorized translation from the German. Edited by S. MacCuen Smith, M. D., Clinical Professor of Otology, Jefferson Medical College, Philadelphia. With 244 colored figures, on thirty-nine lithographic plates and ninety-nine text illustrations. W. B. Saunders & Co., Philadelphia and London. Price, $3.00 net.

In this work the author has beyond question attained his object of supplying practically everything of importance in the elementary study of otology. The text is all that could be desired and, taken together with the wealth of superbly executed illustrations, constitutes by far the best work of the kind that has yet been offered to the profession of this country. The volume is in every respect fully up to the high standard of excellence attained in the other numbers in Saunders' series of hand atlases, which is a sufficient guarantee that it will be of inestimable value both to students as a text-book and to practitioners as a work of reference.

